# How Person-Organization Fit Impacts Employees' Perceptions of Justice and Well-Being

**DOI:** 10.3389/fpsyg.2017.02318

**Published:** 2018-01-09

**Authors:** Marta Roczniewska, Sylwiusz Retowski, E. Tory Higgins

**Affiliations:** ^1^Faculty in Sopot, SWPS University of Social Sciences and Humanities, Sopot, Poland; ^2^Department of Psychology, Columbia University, New York, NY, United States

**Keywords:** regulatory focus, regulatory fit, person-organization fit, procedural justice, job burnout, fairness perception

## Abstract

Regulatory fit theory predicts that when individuals adopt strategies that sustain their motivational orientations, they feel right about what is happening. Our aim was to test these predictions at the person-organization level. Across three studies, we expected and found that a feeling right experience that results from a match between an employee and an organizational climate produces perceptions that the company's prevailing procedures are fair. In Study 1 (*N* = 300), a survey among employees of distinct companies, we observed that the more organizational characteristics matched individual promotion and prevention focus of the employees, the more the employees perceived their workplace as just. Study 2 (*N* = 139), a randomized-control experiment, replicated this pattern by demonstrating that individuals with a predominant promotion focus assigned fairness to the organizational conduct most strongly when they recalled events characterizing a promotion-oriented environment; on the contrary, individuals with a predominant prevention focus deemed their workplace most fair when they were asked to recall prevention-related conduct of their company. In Study 3 (*N* = 376), a cross-sectional field study, we found that regulatory non-fit was associated with lower procedural justice perceptions and this, in turn, related to higher burnout. Theoretical and practical implications of applying regulatory fit theory to person-organization relationships are discussed.

## Introduction

Substantial research on organizational justice attests to people being keenly attuned to matters of justice in their workplace (Brockner, 2016). Notably, many researchers emphasize that fairness is not an objective concept but rather a psychological effect, constructed by the recipient of the procedure (van den Bos, 2005). Thus, recent approaches to organizational justice have focused on the nature of the mental shortcuts used in forming and applying psychological judgments of fairness: the so-called *fairness heuristics* (Cropanzano et al., 2001). In this paper we consider the principle of *regulatory fit* (Higgins, 2000) as another potential contributor to employees' fairness judgments about organizational conduct; specifically, the fit between employee's self-regulatory focus and the regulatory focus of their organization.

When performing tasks at work, individuals have preferred goal-pursuit strategies (e.g., *John prefers to make decisions quickly*) while at the same time they need to deal with the more or less formal rules that have been established in their companies regarding which strategies should be used in goal pursuit (e.g., *John's company urges the employees to be careful and consider different options prior to making a decision in order not to make a mistake*). As in this example, the employee's self-regulatory inclinations may not match their company's codes of conduct regarding how to pursue goals.

Regulatory fit theory argues that when individuals can adopt strategies that sustain their motivational orientations, they *feel right* about what is happening (Higgins, 2005). When they cannot, they *feel wrong* about what is happening (Camacho et al., 2003). We propose that these fit and non-fit experiences that derive from person-organization fit and non-fit, respectively, can produce perceptions that the procedures prevailing in the company are right and fair, or are not right and fair, respectively, which translate into perceptions of procedural justice in that workplace.

The purpose of this paper is two-fold. First, in line with a paradigmatic shift in the organizational justice literature (Brockner et al., 2015), we aim to examine fairness as a consequence rather than as a cause. In doing so, we propose that the experiences of person-organization regulatory fit—*feeling right* from fit and *feeling wrong* from non-fit—are one source of how people perceive and assess fairness and procedural justice in their organizations. This hypothesis will be tested in two studies: Study 1, which is a cross-sectional field study, and Study 2, which is a randomized controlled experiment.

Another aim of this investigation relates to the consequences that such fit and non-fit can have for employee well-being. Given that both individual regulatory focus and organizational rules of conduct are relatively stable, we propose that there is a risk of developing burnout symptoms when the demands in employees' work environment are a non-fit with their personal goal pursuit orientation, exposing them to constant *feeling wrong* experiences about organizational fairness. If so, then justice perceptions could serve as a mediating mechanism between person-organization non-fit and burnout symptoms. This possibility is tested in Study 3, which is a field study.

We consider these assumptions in terms of the self-regulatory orientations and strategic preferences identified by regulatory focus theory. We begin by describing this theory (Higgins, 1997, 2012). Then, we consider the mechanisms of regulatory fit and describe the levels at which it has been tested in the workplace. Finally, we propose the link between person-organization fit and justice perceptions, and describe three studies that tested the proposed relations.

## Employees' regulatory focus orientations and strategic inclinations

Regulatory focus theory (Higgins, 1997, 1998; Higgins and Cornwell, 2016) proposes two basic human concerns in goal pursuit–for growth and for security. The promotion orientation is concerned with growth and advancement, with moving from the current status quo “0” to a better state “+1”. Promotion-focused individuals are attentive to ideals and strive toward their wishes and aspirations. They are sensitive to the presence and absence of positive outcomes; hence, they concentrate on attaining gains (Halamish et al., 2008) and exhibit positive implicit attitudes toward objects that bring them closer to the desired end-state (Roczniewska and Kolanczyk, 2014; Kolańczyk and Roczniewska, 2015). Individuals with a promotion focus prefer eager strategies in their decision making and goal pursuit (Crowe and Higgins, 1997; Higgins, 2000).

In contrast, the prevention focus orientation is concerned with safety and security, with maintaining a satisfactory status quo “0” against a worse state “−1”. Individuals high in prevention are attentive to oughts, duties, and responsibilities (Higgins, 1997). Consequently, they are more interested in maintenance than advancement. Prevention-focused individuals concentrate on avoiding potential losses; they are sensitive to the presence and absence of negative outcomes (Higgins and Tykocinski, 1992). Individuals with a prevention focus prefer vigilant strategies and error monitoring in their decision making and goal pursuit (Crowe and Higgins, 1997; Higgins, 2000).

Empirical findings indicate that promotion and prevention foci are uniquely associated with work-related outcomes (Lanaj et al., 2012). Promotion-oriented employees are likely to engage in work activities because of their ideals and aspirations, whereas employees with a prevention focus perform their work from a sense of duty (Johnson and Chang, 2008). When making work-related decisions, individuals who score high in promotion are concerned with the gains and advancements that the decision might bring, whereas individuals who score high in prevention are more attentive to potential losses (Gamache et al., 2015). Promotion-oriented employees seek chances to develop and gain new experiences at work; they want to express creativity and choose strategies for obtaining desired outcomes freely. In their meta-analysis, Lanaj et al. (2012) demonstrated that a promotion focus is positively related to innovative performance.

In contrast to promotion-oriented employees, prevention-oriented employees focus on completing their duties and obligations correctly. They exhibit a need to follow instructions and scripts of actions. Prevention correlates positively with adhering to work-related rules, such as safety regulations (Wallace and Chen, 2006; Kark et al., 2015). Prevention-oriented employees prefer a transactional leadership style where clear rules exist on how to act to achieve rewards and avoid punishment (Hamstra et al., 2011).

These findings are consistent with promotion and prevention having distinct self-regulatory concerns that are associated with unique strategic preferences and inclinations. What happens, then, when the organizational environment demands a strategy that does not fit with an individual's promotion or prevention focus? In particular, what happens when an organization emphasizes gains and aspirations vs. potential losses and obligations? To address this question, we turn to Regulatory Fit Theory (Higgins, 2000; Higgins et al., 2003).

## Regulatory fit at the person-organization level

Findings from studies testing regulatory focus theory contributed to the development of regulatory fit theory (Higgins, 2000, 2005), although regulatory fit is not restricted to prevention and promotion motivations. The basic idea is that people experience fit when they adopt goal pursuit strategies or engage in activities in a manner that matches their self-regulatory orientation (Higgins, 2000; Avnet and Higgins, 2006). This, in turn, affects the value of choices that are made, the effectiveness of persuasion, and the quality of performance. Three potential mechanisms can contribute to such regulatory fit effects (Higgins, 2012): fluency, strength of engagement, and the experience of *feeling right*.

There is evidence that when a message matches regulatory focus of the message recipient, it is processed more fluently (Lee and Aaker, 2004). Such ease and comprehensibility influence judgments and decisions in line with a *feelings-as-information* mechanism (Schwarz, 2002). Indeed, studies find that the effect of regulatory fit on positive evaluations is mediated via high processing fluency (Lee and Aaker, 2004). Regulatory congruence between a supervisor and an employee is related to a greater ease of interaction (Ritchie, 2009). Engagement in a goal pursuit, including persistence, is also strengthened by regulatory fit. Regulatory fit supports and reinforces engagement because the strategy adopted to execute the task provides a sense of control (Higgins, 2006, 2012). Förster et al. (1998) demonstrated that regulatory fit is related to longer time spent working on a given task and stronger effort put into it. In an organizational context, a fit between an employee's focus and a manager's framing of a statement about organizational changes is associated with better employee adaptation to the changes, and higher employee work engagement at the time of transitions (Petrou et al., 2015). Finally, when there is a fit between one's goal-pursuit orientation and the manner of goal pursuit, the individual *feels right* about what he or she is doing. The experience of regulatory fit validates the goal pursuit process by making a person feel that the way they are pursuing the goal is the *right* way (Avnet et al., 2013). This experience is not merely a pleasant hedonic one. According to Merriam-Webster Collegiate Dictionary (2005), one of the meanings of right is *correct* or *proper*. Feeling right about a situation confirms and supports actions as true, legal, and just. There is evidence that people who experience regulatory fit deem deeds and actions under consideration as being more moral, independent of hedonic mood (Camacho et al., 2003). Fransen and Hoeven (2011) demonstrated that employees perceive a written refusal of their requests as more fair when it is framed in terms that match vs. mismatch their induced regulatory focus. In a study on students' reactions to a tuition increase, Li et al. (2011) found that a fit between induced regulatory focus and message framing was associated with higher levels of perceived justice regarding their university's conduct.

The three mechanisms described above as underlying regulatory fit effects on emotions, judgments, decision-making, and performance have been tested in multiple settings, including marketing (Avnet and Higgins, 2006), management (Stam et al., 2010), health (Latimer et al., 2008), and communications (Webb et al., 2017). Regulatory fit as a phenomenon has also been explored in the area of work and organizational psychology. Most of these studies have been concerned with interpersonal fit (e.g., Ritchie, 2009; Hamstra et al., 2013), task fit (e.g., van Dijk and Kluger, 2011), or message fit (Stam et al., 2010; Li et al., 2011). To our knowledge, no previous study has examined the higher level fit between an employees' regulatory focus and the regulatory focus of their employing organization.

Here, we propose that regulatory focus and fit could apply to organizations and its relation to employee orientations. There is some research that points to the possibility that promotion and prevention strategies can be part of the identity of a group, forming a collective regulatory focus (Faddegon et al., 2008). Sacramento et al. (2013) have found that team-level promotion focus moderates the impact of challenges on group creativity (see also Levine et al., 2000, 2016). Furthermore, cross-cultural studies on over 7,000 individuals from 28 societies showed that when a person's promotion focus is a mismatch with the prevalent personalities of other people in that person's culture, self-esteem, and well-being is lowered at the individual level (Fulmer et al., 2010).

These results point to the possibility that promotion and prevention orientations can be detected on a higher level than just an individual or a team. Interestingly, Johnson et al. (2015, p. 1,520) note that although collective (e.g., team-level) regulatory focus has been demonstrated to exist, and to be linked with multiple outcomes, “a broad climate shared by a large organization remains unstudied.” Our studies address this shortcoming by proposing that the promotion vs. prevention distinction can be used to describe differences in organizational climate as it relates to the systems of prevalent goal pursuit strategies and values of an organization. Promotion-oriented organizational conduct would be associated with innovation, wherein employees are motivated to adapt the strategy of being creative and ready to take risks. The mission and the values underline the need to develop and be oriented toward gains. In contrast, prevention-oriented organizational conduct would emphasize the importance of complying with established rules to avoid losses; hence, it can lead to a high level of standardization of procedures and actions. The motive to avoid any losses would comprise vigilant strategies, sustaining the status quo and minimizing chances for errors.

Although there are many studies on the types and consequences of person-organization fit in general (for a meta-analysis see Kristof-Brown et al., 2005), to the best of our knowledge no published research has examined the issue of regulatory fit between a person and an organization as it relates to an organization's promotion or prevention focus. Yet, this subject is important because organizational cues regarding goal pursuit can sustain or hinder individual motivation to reach organizational aims. We need to understand better how organizational contexts that set standards and strategic preferences affect employees who have particular regulatory foci.

## How “feeling right” from fit can influence perceptions of fairness

In line with a distinction proposed by Colquitt and Rodell (2015), we understand *justice* as “the perceived adherence to rules that reflect appropriateness in decision contexts” (p. 188), whereas *fairness* is more of a broad perception of rightness (Colquitt and Rodell, 2015). In the early phases of theorizing about this concept, Leventhal (1977) argued that the perception of fairness is based on deliberate processing; that is, employees engage their time and effort in considering outcome distribution and the distinct processes that led to it. However, this view has been challenged over the years with studies demonstrating that fairness perceptions can be formed quickly, without much reflection on specific rules or particular events (Colquitt and Zipay, 2015). These judgments may be based on heuristics that use whatever data is available, such as the order of the information (van den Bos et al., 1997), connoted affect (Schwarz, 2002), or processing fluency (Reber et al., 2004).

Of special significance for the present research is the following question: Can fairness perceptions about organization be based on regulatory fit and non-fit experiences? The results of studies by Camacho et al. (2003) suggest that they can. Their research showed that a negative experience from a regulatory non-fit led individuals to feel more guilty about their past sins (Studies 1–2); i.e., *feeling wrong* from non-fit transferred to feeling morally wrong—“If it feels wrong, it *is* wrong.” In addition, regulatory fit increased the perceived morality of another person's actions (Study 3) and the perceived righteousness of a public policy (Study 4)—“If it feels right, it *is* right.” Importantly, these effects were found to be independent of the positivity of the participants' mood. Similarly, Li et al. (2011) found that regulatory fit mitigated the negative reactions to a change in the format of a final examination among business school students. Namely, framing the reasons for change in a manner that was congruent with a previously activated focus led to higher perceptions of fairness regarding the teacher's decision to change the exam format from multiple choice test to essay questions.

In line with the results of these studies, we propose that a match between the regulatory focus of the person and the organizational climate engenders an experience that the company's conduct is right or proper, and non-fit produces an experience of things being done in a wrong or improper way. As described above, the feeling right and feeling wrong experiences are more than just pleasant and unpleasant feelings. They form a sense of rightness or wrongness. In addition, previous studies have shown that these experiences can be transferred when an individual evaluates the morality of actions or events. People do confuse the sources of their experiences, allowing for such transfers (e.g., Schachter and Singer, 1962; Schwarz and Clore, 1983; van den Bos et al., 1997; Reber et al., 2004).

We propose that employees experience what *feels right* in their workplace as *being* right, and what *feels wrong* as *being* wrong, thereby using this heuristic as a basis for their perceptions of fairness. We propose that regulatory fit from a match in strategic inclinations between a person and an organization can contribute to perceptions of fairness in organizational settings. Because regulatory fit relates to the relation between employee's preferred goal pursuit strategies and organizational demands regarding such strategies, i.e., goal pursuit means and procedures, our focus is on *procedural justice* perceptions. The latter is concerned with employee evaluations of organizational means rather than outcomes (Brockner, 2016). Notably, the regulatory fit experience, which occurs during the goal pursuit process, does not depend on the final outcome of the action. Given this, it need not affect distributive justice. Whether the outcome of the goal pursuit itself is positive or negative, regulatory fit can affect perceptions of the *process* that led to the outcome (Higgins, 2012); that is, whether the *manner* of goal pursuit is fair. We propose, therefore, that regulatory fit influences perceptions of procedural justice.

Given this line of reasoning, we propose that person-organization regulatory fit relates to higher procedural justice perceptions. Namely:

*Hypothesis 1*. The more promotion-oriented the organizational climate, the higher the procedural justice perceptions among employees high (vs. low) in promotion focus.

*Hypothesis 2*. The more prevention-oriented the organizational climate, the higher procedural justice perceptions among employees high (vs. low) in prevention focus.

## Study 1

Study 1 was a cross-sectional field research aimed to investigate the consequences of person-organization regulatory fit for procedural justice perception. In line with a feeling right experience, we expected that the more organizational characteristics match individual focus, the more the employee will perceive his or her workplace as just and fair.

### Method

#### Participants

In this study 300 participants from various organizations in Poland filled in paper-and-pencil questionnaires provided by our research assistants. The organizations we surveyed included three local government units, two service companies, and one manufacturing company. The sample consisted of 148 females and 150 males (two participants provided no data on their gender). On average, participants were 35.85 years old (*SD* = 6.95) and had worked for their current organization for 10.33 (*SD* = 5.84) years. Due to missing data in questionnaires administered, we had to exclude 6 participants.

#### Procedure and materials

The study was approved by the departmental review board. Our research assistants distributed the questionnaires to the targeted respondents and sought their consent to complete the survey on a voluntary basis. The first page of the survey informed participants of the purpose of the study and explained that the individual data of the participants would be confidential. The next pages obtained self-ratings concerning: employees' chronic regulatory focus, organizational regulatory focus, and procedural justice perceptions.

##### Employee's regulatory focus

To assess the level of chronic promotion and prevention foci from the perspective of an individual at work, we applied Work Regulatory Focus Scale (Neubert et al., 2008) adapted by Roczniewska et al. (2013). Each scale consists of nine statements rated on a five-point scale ranging from 1 (totally disagree) to 5 (totally agree). The scales were *promotion focus* (e.g., “I take chances at work to maximize my goals for advancement.”; α = 0.83), and *prevention focus* (e.g., “I concentrate on completing my work tasks correctly to increase my job security.”; α = 0.79).

##### Regulatory foci of the organizational climate

Organizational regulatory foci levels were measured using a newly developed unpublished scale that allows assessment of organizational climate with respect to two foci: promotion (e.g., “This company provides employees with opportunities to develop”; α = 0.82) and prevention (e.g., “This company often controls how employees perform their duties”; α = 0.78). The scale consists of 10 items (5 for promotion and 5 for prevention) rated on a five-point scale from 1 (strongly disagree) to 5 (strongly agree). More information on the validity process can be found in the Appendix A.

##### Procedural justice

The perceived fairness of organizational procedures was measured using a subscale from the organizational justice measure developed and validated by Colquitt (2001) in Polish adaptation performed by Retowski et al. (2003). The subscale consists of 7 items (e.g., “Do those procedures uphold ethical and moral standards?”). Individuals are asked to answer using a scale ranging from 1 (to a very small extent) to 5 (to a very large extent). The Cronbach's alpha coefficient for the scale's reliability was α = 0.87.

### Results and discussion

Means, standard deviations, and zero-order correlations between study variables are displayed in Appendix B (Table B1). The aim of the analysis was to demonstrate the influence of an interaction between individual and organizational regulatory focus on procedural justice perceptions. We administered a simple moderation analysis (Model 1; Hayes, 2013) with organizational focus serving as a predictor (X), individual regulatory focus as a moderator (M), and procedural justice as a dependent variable. Two separate analyses were conducted: one for promotion fit (juxtaposing individual and organizational promotion foci) and another for prevention fit (juxtaposing individual and organizational prevention foci).

We performed the statistical analysis using the SPSS 24 statistical package with PROCESS macro (Hayes, 2013). To estimate the significance of the moderation effect, we used Bootstrapping 10,000 with 95% confidence bias-corrected intervals (MacKinnon et al., 2004; Preacher and Hayes, 2004). The assumptions are that the effects are considered significant when the average estimation values are within the 95% confidence interval (CI), so that the CI does not include zero.

To investigate the influence of the moderator on the relation between the predictor and the dependent variable, we used the Johnson-Neyman (N-J) technique (Johnson and Fay, 1950). This method was chosen because the moderator is a continuous variable, and the use of arbitrary values above and below one standard deviation from the average raises concerns (Preacher et al., 2006). This technique allows to indicate quantitative values of the moderator (called *significance regions*), where the influence of the predictor on the dependent variable is significant. The so-called J-N points are exact values of the moderator, where the influence of the independent variable on the dependent variable changes (from insignificant to significant, or vice versa). These points define the significance regions. The J-N points and significance regions are calculated based on the confidence intervals calculated for each moderator value. If the 95% CI does not contain 0, then the influence of the independent variable on the dependent variable at a given moderator level is significant at *p* < 0.05 level (Hayes, 2013). In line with recommendations (Hayes, 2013), we present unstandardized correlation coefficients (B) in Tables 1A,B.

**Table 1A:** Regression analysis of employee promotion, organizational promotion, and their interaction term on justice perceptions in study 1.

	***B***	***SE***	***t***	***p***	**LL**	**UL**
*Intercept*	2.90	0.61	4.74	0.00	1.70	4.11
Employee promotion	−0.31	0.16	−1.90	0.06	−0.63	0.01
Organizational promotion	−0.09	0.18	−0.49	0.62	−0.45	0.27
Interaction (Employee promotion × Organizational promotion)	0.14	0.05	3.05	0.00	0.05	0.24

**Table 1B:** Regression analysis of employee prevention, organizational prevention, and their interaction term on justice perceptions in study 1.

	***B***	***SE***	***t***	***p***	**LL**	**UL**
*Intercept*	7.12	1.56	4.55	0.00	4.04	10.20
Employee prevention	−1.35	0.37	−3.67	0.00	−2.07	−0.63
Organizational prevention	−0.87	0.41	−2.12	0.04	−0.68	−0.06
Interaction (Employee prevention × Organizational prevention)	0.32	0.09	3.39	0.00	0.14	0.51

*n = 10,000 bootstrapping resamples; B, unstandardized coefficient; SE, standard error; LL CI, lower level; UL CI, upper level of bias corrected bootstrapped confidence intervals for α = 0.05*.

To test hypotheses 1 and 2, moderation analyses both for promotion fit and prevention fit were conducted and are depicted in Tables 1A,B. As Tables 1A,B show, the interactions between individual and organizational regulatory foci were significantly associated with procedural justice perceptions for both promotion and prevention fit.

#### Promotion fit

**Figure 1:**
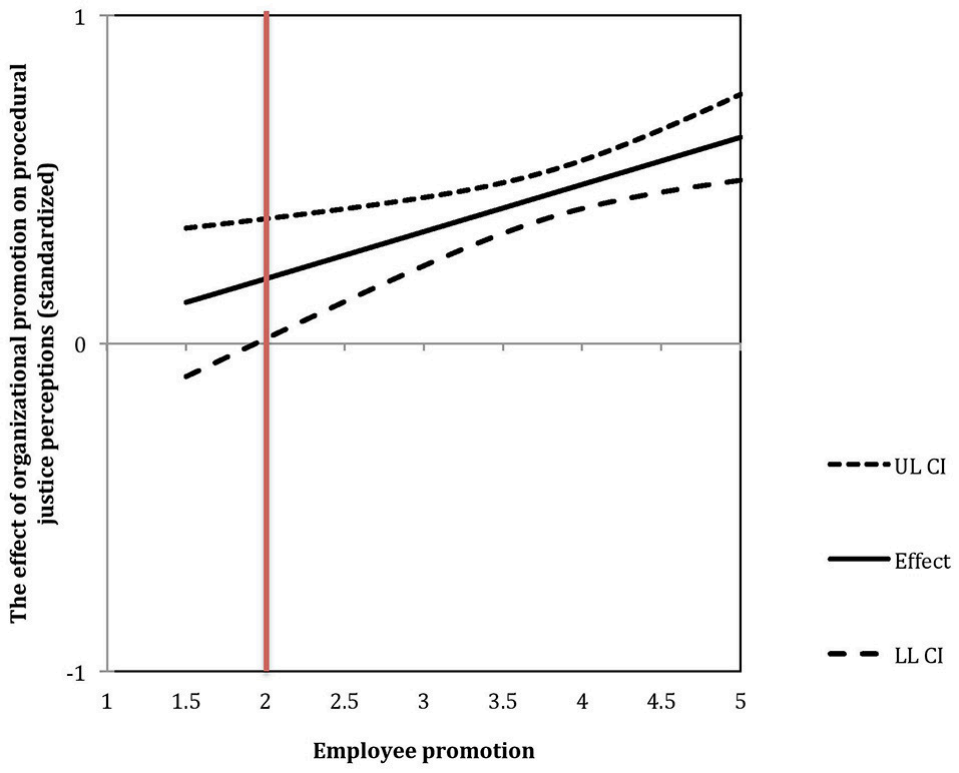
The effect of organizational promotion on procedural justice perceptions moderated by employee promotion in Study 1. Vertical red line indicates the Johnson-Neyman point.

The interaction between individual and organizational promotion is responsible for a significant increase in the predictive value of the model by 2%, *F*(1, 293) = 9.31, *p* < 0.001. In order to understand the meaning of interactions, we have analyzed the moderation using the Johnson-Neyman procedure (Johnson and Fay, 1950). The results of the analysis are illustrated in Figure 1. It represents the strength of the relation between the promotion of the organization's climate and procedural justice perceptions depending on the employee's promotion level. The J-N point (marked with a vertical red line) is at 1.94 points of the employee's promotion. This means that for people who scored 1.94 and more points on the promotion focus scale (1–5 scale), the more promotion-focused their company is, the more fairness they perceive in its conduct. For people who have scored relatively low on promotion focus (below 1.94), there is no link between the organization's promotion and its justice perceptions. This result is in line with Hypothesis 1.

#### Prevention fit

**Figure 2:**
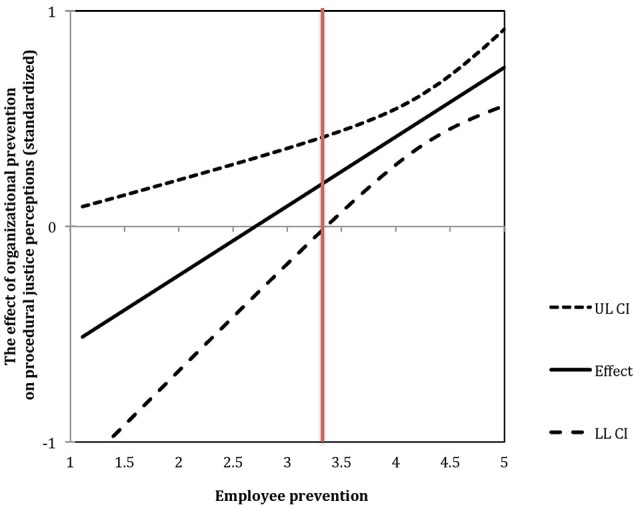
The effect of organizational prevention on procedural justice perceptions moderated by employee prevention in Study 1. Vertical red line indicates the Johnson-Neyman point.

The interaction between individual and organizational prevention enhanced the amount of explained variance in procedural justice perceptions by 3%, *F*(1, 293)= 11.51, *p* < 0.001. Again, we have analyzed the moderation using the J-N procedure (Johnson and Fay, 1950). The results of the analysis are depicted in Figure 2. It demonstrates the strength of the relationship between the prevention of the organization's climate and procedural justice perceptions depending on the employee's prevention level. The J-N point (marked with a vertical red line) is at 3.36 points of the employee's prevention. The results indicate that, in line with Hypothesis 2, only for employees who have scored relatively high on prevention focus (above 3.36 on a 1–5 scale), there is a positive link between the organization's prevention and its justice perceptions. Meanwhile, for people who scored lower, the relation ceases to exist.

The results of Study 1 are in line with our predictions, demonstrating a transfer of value from regulatory fit to moral judgments regarding organizational conduct. Employees with relatively strong promotion focus need strategic eagerness in their organization's climate: moving forward, maximizing gains, focusing on development, and pursuing innovation. The more the company they are employed in possesses these eager characteristics, the more they perceive its procedures as just. Similarly, individuals with relatively strong prevention focus prefer strategic vigilance in their organization's climate: maintenance, minimizing losses, focusing on safety, and adhering to procedures. For them, organizations and their conduct seem more fair the more they posses these vigilant features. Overall, these results are consistent with previous research (Camacho et al., 2003) showing that feeling right produced by regulatory fit is transferred to perceiving something as morally right (here: company's procedures). This research extends previous findings by showing that *the more* the organization possesses strategic characteristics that fit employees' regulatory focus, the *more justice* is perceived in the company's procedures. These findings demonstrate that employees are sensitive to the *degree* of match between their strategic preferences and what the organizational climate has to offer.

Study 1 examined for the first time the consequences of regulatory fit for a higher level of than it had ever been previously framed; namely, the fit between the employees' strength of regulatory focus and the organization's regulatory focus strategic emphasis. Previously described strategic characteristics that fit each focus (Lee and Higgins, 2009) were used to describe organizational climate. The study found that strategic emphasis can be detected at an organizational level, and that they interact with employees' strength of regulatory focus. As predicted, an organization that emphasizes gains, maximization, and advancement fits employees with a stronger promotion focus, and an organization that emphasizes loss aversion, maintenance, and safety fits employees with a stronger prevention focus, and fit enhances perception of the organization's procedural justice.

## Study 2

Study 1 has the advantage of testing our hypotheses across a relatively broad range of organizations, including local government units, service companies, and a manufacturing company. The disadvantage of this cross-sectional study is that it precludes causal inferences, even though there are well-documented experimental studies on the influence of regulatory fit on moral judgments (e.g., Camacho et al., 2003; Li et al., 2011). To address this limitation, Study 2 was designed to experimentally manipulate regulatory fit by activating the participants' idea that a company had either a promotion-related or prevention-related strategic emphasis. Additionally, to strengthen the internal validity of the effect (cf. Wojciszke, 2004), we used *predominant focus* instead of two regulatory foci as separate variables (see Molden and Higgins, 2004; Cesario and Higgins, 2008). We expected the effects to be stronger for individuals with greater predominance in one focus than the other. Finally, for the purpose of higher external validity (cf. Wojciszke, 2004), we used a different justice perception instrument.

In line with previous hypotheses and Study 1's findings we anticipated that:

*Hypothesis 3*. For individuals with predominant promotion focus, higher perceptions of their organization's justice would occur when recalling promotion-related organizational conduct compared to prevention-related conduct or control events.

*Hypothesis 4*. For individuals with predominant prevention focus, higher perceptions of their organization's justice would occur when recalling prevention-related organizational conduct compared to promotion-related conduct or control events.

### Method

#### Participants

The data was gathered with an online survey implemented in Qualtrics. Respondents were 311 Polish employees within different occupational sectors recruited with network sampling by 10 research assistants (see Demerouti and Rispens, 2014). After excluding 10 participants who failed to provide any responses to open-ended questions and another 170 whose responses did not meet task criteria (see Organizational Regulatory Focus Manipulation), 139 respondents (108 women, 31 men) formed the final sample for the analysis. The mean age of the participants was 41.92 years (*SD* = 9.58). On average, participants worked for their present employer for 6 years.

#### Procedure and materials

The first page of the survey informed participants that the purpose of the study was to examine workplace attitudes and behaviors. The instruction also explained that the individual data of the participants would be confidential, and asked the participants to be genuine in their responses. The procedure started with an organizational regulatory focus manipulation, which prompted participants to describe relevant situations that took place in their company (see below). Next, individuals were asked to fill in two scales (see below): organizational justice perception instrument, and work regulatory focus measurement. We also gathered demographic data. The instruments were implemented into Qualtrics software which randomly assigned participants to one out of three conditions based on organizational regulatory focus manipulation: promotion, prevention, and control. Before conducting the study, we received departmental review board approval.

##### Organizational regulatory focus manipulation

Individuals were randomly assigned to one of the three experimental groups: organizational promotion, organizational prevention, and control condition. They were asked to recall and briefly describe three events that took place in their workplace. The items for promotion- and prevention-related situations were based on the Organizational Regulatory Focus Scales that emphasize promotion or prevention events that take place in the workplace (Roczniewska et al., 2016). In the manipulated *promotion-related* organizational climate, the three events were: (1) promoting creativity among employees; (2) providing opportunities for their growth; and (3) encouraging pursuing ideals and aspirations among the workforce. In the manipulated *prevention-related* organizational climate, the three events were: (1) undertaking actions to avoid losses; (2) preventing employees from making mistakes; and (3) monitoring fulfillment of the duties and responsibilities. The situations for control condition were chosen from a list of seven events that have been rated beforehand by competent judges (*N* = 20) for promotion and prevention. We chose three situations that were evaluated as low in both foci. In the control group, the three events were: (1) the last staff-meeting subject and its course; (2) actions undertaken to integrate employees; and (3) ecology-related activities in their workplace. The participants in each condition were asked to provide descriptions for all three events. This general technique for inducing regulatory focus by having participants recall certain events from the past has been previously validated in numerous studies (e.g., Higgins et al., 1994; Liberman et al., 2001; Cesario et al., 2004).

##### Organizational justice

For the purpose of higher external validity (Wojciszke, 2004), we used a different justice perception instrument. The perceived fairness of organizational conduct was measured using a newly-developed scale that consists of 5 items (e.g., “I consider the conduct in my workplace fair”; Roczniewska, 2016). Participants are asked to answer using a scale ranging from 1 (definitely not) to 5 (definitely yes). The Cronbach's alpha coefficient for the scale's reliability was α = 0.95. In validation studies this instrument correlated highly with Colquitt's organizational justice scales (*r* = *0*.84, *p* < 0.001), supporting its validity (Roczniewska, 2016).

##### Employee's self-regulatory focus

Similarly to Study 1, we applied Work Regulatory Focus Scale (Neubert et al., 2008) to measure the level of chronic promotion (α = 0.81) and prevention (α = 0.81) foci from the perspective of an individual. By subtracting each individual's prevention focus score from that individual's promotion score, we obtained a continuous *focus* variable with values ranging from −2.11 to 2.67 (*M* = −0.34, *SD* = 0.67). Values below 0 indicate a predominant prevention focus, whereas values above 0 indicate a predominant promotion focus (see, for example, Cesario and Higgins, 2008).

### Results and discussion

#### Manipulation control

First, two independent judges analyzed the responses with respect to whether they generally adhered to the task, and excluded responses that were blank or did not follow the task^1^. This resulted in 614 correct responses. Because each participant was asked to respond to three questions, we only kept individuals who provided three task-related answers. The reason for this strict criterion of providing all three task-related answers is the evidence that if people find it difficult to provide the requested number of examples they are likely to conclude that this is because such cases are infrequent, which would defeat the purpose of the experimental manipulation (see, for example, Schwarz et al., 1991). The final sample was *n* = 139, with 46 individuals in manipulated promotion condition, 47 individuals in manipulated prevention condition, and 46 in control condition.

Next, we trained 18 competent judges in Regulatory Focus Theory (Higgins, 1997, 2012). The judges were randomly assigned with answers from all three experimental groups to evaluate. Nine of the judges were asked to rate to what extent each of the responses depicted a promotion-focused organizational climate; the other nine followed the same task with regards to a prevention-focused organizational climate. They used a 5-point scale from 1–*totally uncharacteristic of promotion (prevention)* to 5–*strongly characteristic of promotion (prevention)*. The judges were unaware of participants' experimental conditions. To calculate agreement among rates we used a non-parametric test called Kendall's *W* (Kendall's coefficient of concordance; Corder and Foreman, 2011). Higher values of *W* indicate a greater degree of unanimity among responders. The mean agreement for promotion judges was *W* = 0.75, whereas the mean agreement for prevention judges was *W* = 0.72. Each response received a mean promotion rating and a mean prevention rating.

To check on the manipulation, we performed a 3 (group: promotion, prevention, control) by 2 (judges ratings: promotion, prevention) repeated-measures ANOVA for the remaining responses. The analysis yielded a significant interaction effect, *F*(2, 136) = 169.15, *p* < 0.001, η^2^ = 0.63. The *post-hoc* comparisons demonstrated that judges' promotion ratings were highest in promotion group (*M* = 3.73, *SD* = 0.60) as compared to prevention (*M* = 2.11, *SD* = 0.57) and control groups (*M* = 1.65, *SD* = 0.44), whereas prevention ratings were highest in prevention (*M* = 3.99, *SD* = 0.56) group as compared to promotion (*M* = 3.42, *SD* = 0.72) and control groups (*M* = 2.29, *SD* = 0.62)^2^.

#### Hypotheses testing

Again, we administered a simple moderation analysis (Model 1; Hayes, 2013). Organizational focus served as a multicategorical predictor (X), coded in the following way: 0–control, 1–prevention, and 2–promotion. Self-rated organizational justice acted as a dependent variable (Y). We used the self-regulatory focus variable as a moderator (M), with values below 0 indicating a predominance of prevention over promotion, and values above 0 indicating a predominance of promotion over prevention. We performed the statistical analysis using the SPSS 24 statistical package with PROCESS macro allowing for multicategorical predictor (Hayes, 2013). Because the J-N procedure is unavailable when multicategorical predictor is involved, we sought to investigate the conditional effects on distinct levels of self-regulatory focus variable (i.e., at the 10, 25, 50, 75, and 90th percentiles, as provided in PROCESS). Conditional effects based on percentiles are in line with simple slope analyses (Cohen et al., 2003), but this procedure guarantees that all quantile values are within the range of the observed data, even if the distribution is skewed (Hayes, 2013), as in this case. In line with recommendations (Hayes, 2013), we present unstandardized correlation coefficients (B) in the text below.

**Figure 3:**
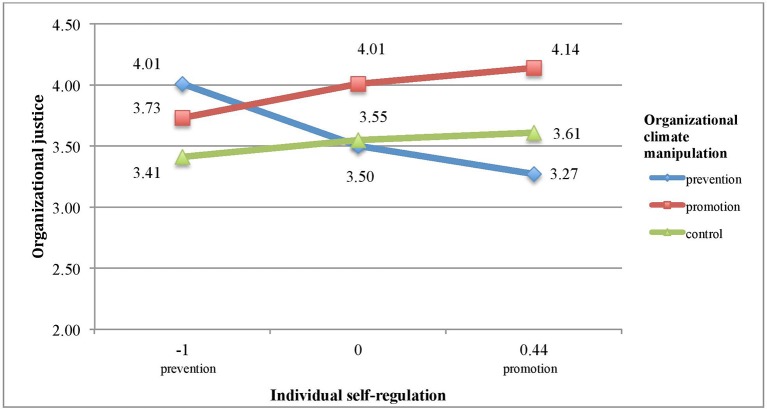
The effect of organizational climate manipulation on justice perceptions moderated by individual self-regulation in Study 2.

The model we tested was statistically significant, *F*(5, 133) = 2.41, *p* = 0.04. It explains 7% of variance in justice perceptions. R-square increase by 3.2% due to interaction term is marginally significant, *F*(2, 133) = 2.31, *p* = 0.10. Figure 3 represents the influence of organizational regulatory focus on justice perceptions on distinct self-regulatory focus levels. Values below zero indicate predominant prevention focus, whereas values above zero are indicative of predominant promotion focus. For the purpose of testing the hypotheses, we used extreme moderator values (10 and 90th percentiles) as indicative of (respectively) prevention and promotion predominance, and compared it with group with balance in both foci.

In line with Hypothesis 3, for individuals with a predominant promotion focus (M_focus_ = +0.44; 90th percentile), recalling promotion-oriented organizational events increased justice perceptions as compared to control (b = 0.5212, SE = 0.2770, LL CI^3^ = −0.0267, UL CI^4^ = 1.0690)^5^ and prevention condition (b = 0.8664, SE = 0.3355, LL CI = 0.2027, UL CI = 1.5300), with no difference between the latter two conditions (b = 0.3452, SE = 0.3249, LL CI = −0.2975, UL CI = 0.9879). 3. In line with Hypothesis 4, for individuals with a predominant prevention focus (M_focus_ = −1.00; 10th percentile) recalling prevention-related organizational events increased justice perceptions as compared to control (b = 0.5932, SE = 0.3280, LL CI = −0.055, UL CI = 1.2419)^6^ and promotion condition (b = 0.2729, SE = 0.3381, LL CI = −0.3959, UL CI = 0.9417), with no difference between the latter two conditions (b = 0.3203, SE = 0.2848, LL CI = −0.2431, UL CI = 0.8837).

The results of Study 2 are in line with our predictions. Specifically, individuals with a predominant promotion focus assign fairness to the organizational conduct most strongly when they recall events characterizing a promotion-oriented environment: supporting innovation, providing development opportunities, and encouraging pursuing ideals among the workforce. On the contrary, individuals with a predominant prevention focus deem their workplace most fair when they are asked to recall prevention-related conduct of their company: what the firm does to avoid losses, prevent mistakes, or how it monitors whether employees fulfill their duties and responsibilities. When the overall pattern is considered with the control condition in mind, Study 2 found a fit effect on increasing perceptions of justice rather than a non-fit effect on decreasing perceptions of justice.

The pattern of results obtained in Study 2 corroborates our previous finding, demonstrating a more causal (given the experimental design of the study) relation between regulatory fit at person-organization level and justice perceptions. These findings are generally consistent with the literature showing that the psychological judgments of fairness may be based on heuristics. Weiss and Cropanzano (1996) argue that if the object of an attitude is abstract, people tend to base their judgment on heuristic thinking. Being a rather abstract concept, fairness may be such a case. Here, we demonstrate that perceptions of organizational justice may be influenced by the *feeling right* experience that regulatory fit creates.

Study 1 demonstrated that regulatory fit with chronic characteristics of organizational promotion or prevention climate can increase perceptions of procedural justice. Study 2 extended these findings by showing that it is not necessary for these features to be stable to experience regulatory fit benefits. Namely, by temporarily activating certain organizational focus we found that even momentary fit produces feeling right experience transferred to justice evaluations. This result provides converging evidence for our predictions and is congruent with previous findings on inducing regulatory fit (cf. Cesario et al., 2008).

## Study 3

Studies 1 and 2 found that feeling right produced by regulatory fit between the employee and the organization is transferred to perceiving the company and its conduct as morally right and just. The purpose of Study 3 was to extend these findings by demonstrating that person-organization regulatory fit and resulting feeling right experience affect employee well-being. The literature provides grounds for this line of reasoning. Studies reveal a beneficial effect of person-culture match on positive psychological outcomes, such as self-esteem, happiness, and positive emotions (Fulmer et al., 2010). Lafrenière et al. (2016) found that promotion-oriented individuals experience higher life satisfaction when they pursue self-enhancement, whereas prevention-focused individuals are happier when they engage in self-protection. Microinterventions targeting regulatory focus (inducing regulatory fit and misfit) have been demonstrated to reduce dysphoric and anxious mood (Strauman et al., 2015).

Here, we aim to expand the relation between regulatory fit and well-being to the organizational context. Importantly, both work environment climate and individual's regulatory focus are relatively stable (Hmieleski and Baron, 2008). Thus, the strain resulting from person-organization non-fit and injustice that stems from it is likely to persist and accumulate over time. Bakker and Costa (2014, p. 114) argue that a lack of fit between personality and job demands can lead to *job burnout*, especially when “employees are often exposed to demands that do not fit with their skills and preferences.” Job burnout is a syndrome described as a state of mental and physical exhaustion resulting from one's professional life (Freudenberger, 1974). Although the early emphasis was on human services, numerous studies later accounted for its presence amongst other professions (e.g., Leiter and Schaufeli, 1996). The Job Demands-Resources model (JD-R; Demerouti et al., 2001) posits that burnout is a combination of exhaustion and disengagement that result from too high demands as compared to resources available to deal with the strain.

In their work Leiter and Maslach (2004) emphasize the importance of an interactive approach to examining the process of burnout development. These authors point to person-environment fit as a proper framework for such investigation. We propose that a lack of match between one's regulatory focus and what the organization emphasizes with regards to goal pursuit focus affects employee well-being by creating a significant source of exhaustion. This would happen not only because organizational demands are incongruent with employee's resources, but because regulatory non-fit engenders the feeling wrong experience that produces perceived unfairness in the workplace. There are grounds to believe that perceived unfairness in the workplace relates to job burnout (Liljegren and Ekberg, 2009). Therefore, we expect that regulatory non-fit is related to stronger burnout, and this relation is mediated via low procedural justice perceptions.

The results of Study 1 and 2 showed that person-organization regulatory fit is linked to how individuals assess justice in their companies, and this pattern was found for promotion and prevention. Hence, in this study we predicted the following general hypothesis:

*Hypothesis 5*. Regulatory non-fit at person-organization level is linked with higher job burnout.

*Hypothesis 6*. The relation between regulatory non-fit at person-organization level and job burnout is mediated via procedural justice perceptions.

### Method

#### Participants

The participants in this study were recruited online via advertisements in social media, science websites, and mailing to professional organizations (e.g., nurses society). In total 377 participants (325 female, 52 male) from various organizations filled in online questionnaires implemented in Google forms. The average age of the participants was 37.28 years (*SD* = 9.79). Participants worked on average 40.71 (*SD* = 11.99) hours a week and on average 7.59 (*SD* = 8.27) years for their current organization.

#### Procedure and materials

Questionnaires were administered online and participants received the link to the Google forms survey. The first page of the survey informed participants of the purpose of the study and explained that the individual data of the participants would be confidential. The next pages acquired self-ratings concerning employee and organizational regulatory focus, and procedural justice perceptions that we have used in Study 1. An additional questionnaire was administered to examine burnout syndromes.

##### Job burnout

To study burnout syndromes, we used the Polish adaptation (Baka and Cieślak, 2010) of the Oldenburg Burnout Inventory (OLBI) which consists of two subscales: exhaustion (e.g., “During my work, I often feel emotionally drained”; α = 0.81) and disengagement (e.g., “Over time, one can become disconnected from this type of work”; α = 0.79) continua (Demerouti et al., 2003). The answering categories range from (1) “strongly agree” to (4) “strongly disagree.”

### Results and discussion

Means, standard deviations, and zero-order correlations between study variables are displayed in Appendix C (Table C1).

#### Fit calculation

Similarly to Study 2's procedure, we calculated *individual focus* by subtracting individual prevention focus from individual promotion focus. Next, we computed *organizational focus* by subtracting organizational prevention from organizational promotion. In both cases scores above 0 indicate a predominant promotion focus, while scores below 0 are indicative of a predominant prevention focus. Hence, we ascribed *fit* condition when both results were high (i.e., promotion-predominant person in promotion-predominant environment) or when both results were low (i.e., prevention-predominant person in prevention-predominant environment). *Non-fit*, on the other hand, occurred when the situation was reverse, i.e., a promotion-predominant person was employed in a prevention-predominant environment or a prevention-predominant person was employed in a promotion-predominant environment. This procedure resulted in 192 participants in *fit* condition, 130 participants in *non-fit* condition, and 55 individuals were excluded from the analysis because either the individual or the organizational focus was 0, thus, indicating a balance rather than a predomination of one focus over the other.

#### Hypotheses testing

The purpose of the statistical analysis was to show the mediating role of procedural justice perceptions in the relation between regulatory fit at person-organization level and job burnout. This relationship was tested in a mediation design. Regulatory fit served as a predictor (X), coded in the following way: 0–regulatory fit, and 1–regulatory non-fit. We used procedural justice as a mediator (Me), and job burnout as dependent variable (Y). Analyzes were carried out separately for the 2 dimensions of burnout: exhaustion and disengagement.

To test for expected mediations we conducted statistical analyses using the SPSS 24 statistical package with the PROCESS macro developed by Hayes (2013). We administered model 4: simple mediation. We used Bootstrapping 10,000 with bias-corrected confidence intervals (MacKinnon et al., 2004; Preacher and Hayes, 2004) for estimating indirect effects and moderated mediation indices. In line with Hayes's guidelines Hayes (2013), unstandardized correlation coefficients (B) are presented in the text and figures.

**Figure 4:**
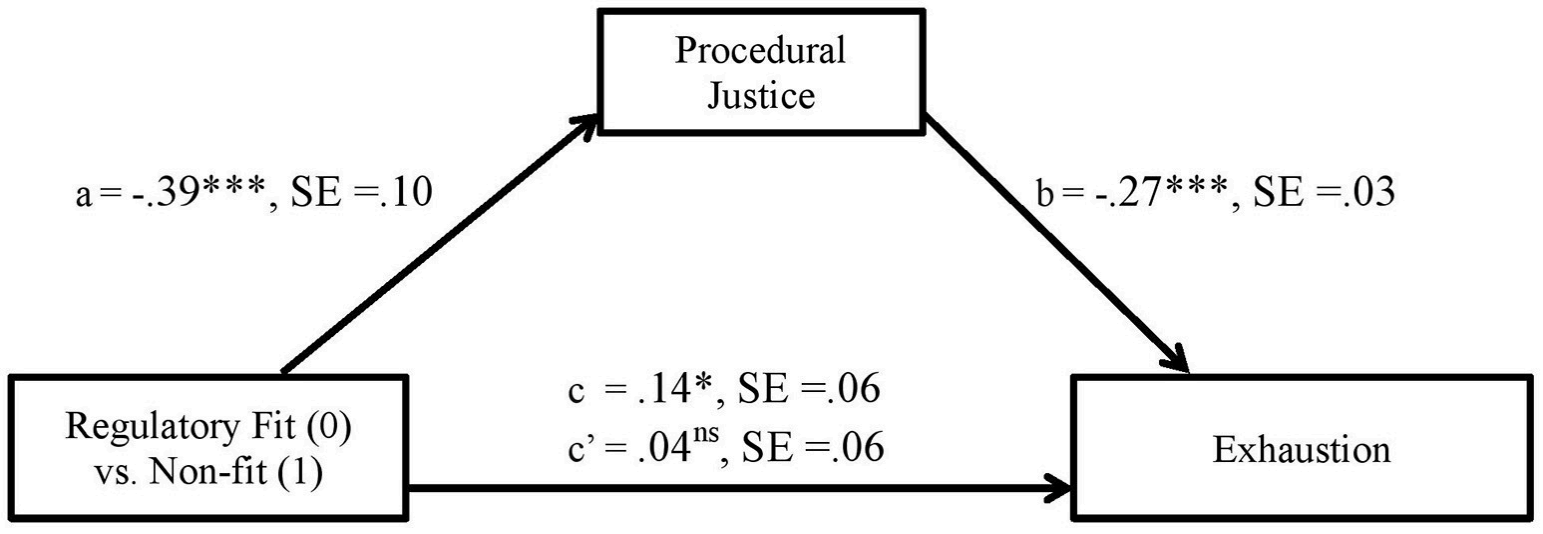
The effect of person-organization regulatory non-fit on exhaustion as mediated via procedural justice in Study 3 (unstandardized coefficients).

**Figure 5:**
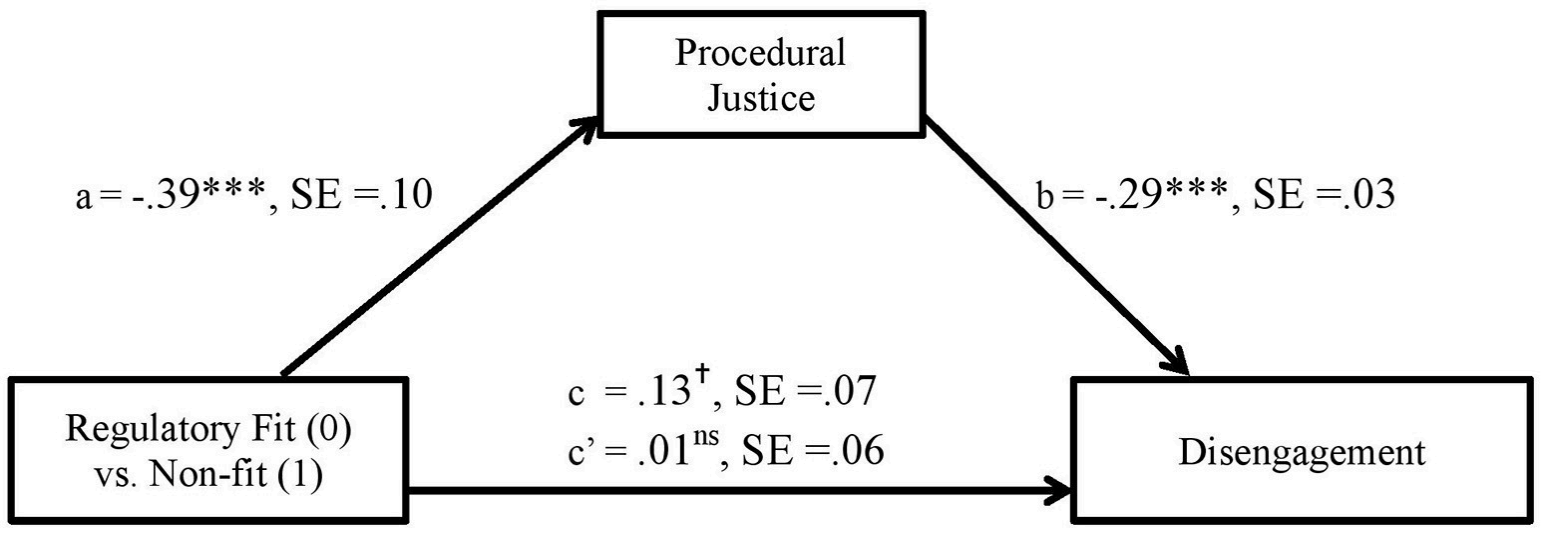
The effect of person-organization regulatory non-fit on disengagement as mediated via procedural justice in Study 3 (unstandardized coefficients).

Figures 4, 5 show unstandardized B coefficients for the paths in mediation models explaining exhaustion and job disengagement, respectively.

The total effect of regulatory non-fit on exhaustion is positive and significant (PE [Point Estimate] = 0.14, SE = 0.06), i.e., non-fit is linked with higher emotional and physical fatigue. Whereas the direct relation between these variables is not statistically significant (PE = 0.04, SE = 0.06), the indirect effect of procedural justice in the relation between non-fit and exhaustion is significant, as expected (Indirect = −0.1038, SE = 0.0296) and comprises 74% of the total effect.

The total effect of regulatory non-fit on disengagement is positive and close to significance (PE = 0.13, SE = 0.07^7^), i.e., non-fit relates to stronger withdrawal from work. Again, whereas the direct relation between these variables is not statistically significant (PE = 0.01, SE = 0.06), the indirect effect of procedural justice in the relation between non-fit and disengagement is significant, as expected (Indirect = −0.1141, SE = 0.0308) and comprises 90% of the total effect. These results indicate that, as expected, the relations between regulatory non-fit and both facets of burnout are mediated via lower procedural justice perceptions.

In sum, the findings obtained in Study 3 replicate the pattern of results observed in Studies 1 and 2, demonstrating that regulatory fit and non-fit are linked with how employees perceive the fairness of company's conduct. Here, we aimed to extend these findings by demonstrating that misfit can contribute to burnout through perceived unfairness of the workplace. We show that when organizational climate does not fit well with individual focus employees suffer more severely from burnout, i.e., have more mental and physical symptoms of exhaustion, and they disengage from their work more frequently. This effect is explained via lower justice perceptions resulting from non-fit. This finding is consistent with other research demonstrating the undesirable consequences of low justice perceptions in organizations (e.g., Lambert et al., 2007).

To our knowledge, only one published paper examined the link between regulatory fit and well-being in organizational settings: Petrou et al. (2015; Study 3) demonstrated that prevention regulatory fit was associated with lower employee exhaustion and higher employee work engagement during organizational change. However, in this particular study regulatory fit related to message-framing, i.e., managerial change communication. Here, we expand this perspective to regulatory fit at the person-organization level, demonstrating that regulatory cues present in organizational climate significantly affect employee well-being by means of creating *feeling right* or *feeling wrong* experiences. This perspective is important because organizational climate affects employees on an everyday basis, dictating proper means of goal pursuit or acceptable standards. If these principles differ from individual's preferences, the context disrupts motivation and negatively affects employee well-being. Our study adds to the body of research describing factors that predict job burnout, and is consistent with other models that point to person-organization misfit as sources of burnout (Leiter and Maslach, 2004; Siegall and McDonald, 2004; Tong et al., 2015).

## General discussion

### Contribution

Drawing on past research on regulatory fit, we proposed that a new level of regulatory fit—the one between an employee's regulatory focus and the organizational climate for goal pursuit—has significant implications for how individuals perceive procedural justice in their workplace. Namely, we demonstrate across three studies that when there is congruence with respect to strategic inclinations (promotion–eagerness, prevention–vigilance) between individuals and what companies demand from them, employees perceive organizational conduct as more fair. The present research also extends the literature on regulatory fit by examining the consequences of the *degree* of the regulatory fit. Namely, Study 1 found that that the *more* the company posses characteristics that are congruent with individual's focus, the *higher* are employees' perception of the company's procedural justice. This enriches regulatory fit research by showing that individuals may be sensitive to the degree the environment fits them, which further allows to build new hypotheses and apply more complex research models.

By experimentally manipulating regulatory fit at the person-organization level in Study 2, we demonstrated that perceptions of fairness can be significantly influenced by contextual factors. The fact that fit can be manipulated provides opportunities for increasing employee fairness perceptions by focusing their attention on organizational conduct that matches their motivational orientation. Just as individuals typically possess both promotion and prevention orientations (even if one predominates), company's' climate and strategic preferences often include both foci (even if one predominates). Given this, individuals' attention can be directed to promotion or prevention aspects of their company's environment in order to create a fit. Interestingly, studies point to the fact that perceived fit may have stronger effects for organizational outcomes than objective fit (see metaanalysis: Verquer et al., 2003).

This research also contributes to the body of knowledge on job burnout antecedents. In Study 3 we observed that regulatory non-fit relates to more frequent burnout symptoms reported by the employees. This is characterized by higher mental and emotional fatigue and stronger disengagement from every-day tasks that occur because non-fit produces a high-demand, stressful situation: feeling wrong about organizational procedures and standards. To the best of our knowledge, no previous study has linked regulatory non-fit with job burnout. Yet, this relationship is important because self-regulation contributes to successful goal pursuit; the lack of fit between individual's needs and organizational demands is associated with perceptions that the situation is morally wrong. This is consistent with affective events theory (Weiss and Cropanzano, 1996), which predicts that employees consider justice issues because just and unjust situations are helpful or harmful, respectively, to goal pursuit progress. Our research links justice perceptions derived from fairness heuristics to psychological symptoms of exhaustion.

Our studies support the notion that feeling right from fit, and feeling wrong from non-fit, can transfer to moral judgments, consistent with the findings of Camacho et al. (2003). This research provides support for their analysis and extends it to the case of actual employees in real organizational settings, demonstrating ecological validity for regulatory fit theory. Our results suggest that the experiences of regulatory fit and non-fit can function as fairness heuristics, explaining why employees can have different evaluative judgments of fairness in the same organization. In that vein, our findings constitute another exemplar of the notion that fairness is in the eye of the beholder. This research points to the importance of the *interactive* nature of the relation between an employee's regulatory system and the manner of goal pursuit outlined in organizational climate. The studies are in line with the “fifth wave” in justice research (Brockner et al., 2015) concentrating on the antecedents of perceiving procedural justice in the workplace.

Overall, the studies presented in this paper point to important consequences of regulatory fit and non-fit at the person-organization level. This research contributes to the literature by demonstrating how regulatory focus congruence between employees and the organizational climate is yet another kind of person-organization fit: one that relates to regulatory standards and strategic preferences.

### Limitations and future research

Despite these contributions to the literature on regulatory fit and organizational justice, limitations of our studies also need to be mentioned. First, the measures we administered in these studies were based on self-reports, resulting potentially in common method bias. Different response categories were used to lower the plausibility of method biases as an explanation for the relations observed in our studies (Podsakoff et al., 2003). Another limitation is that employees may be prone to report a stronger similarity between the situation and their preferences (Petrou et al., 2015), resulting in a possibility of untrustworthy assessment of organizational conducts in Studies 1 and 3. Future studies could, therefore, provide organizational climate ratings from colleagues or managers to add another data source. It should also be noted that to the extent that there was a bias to perceive a fit between personal and organizational focus, it would not in itself account for the non-fit effect on perceived unfairness that, in turn, mediates burnout.

Moreover, the use of a strict inclusion filter in Study 2 resulted in a relatively small sample size. Although necessitated by prior studies showing the effects on judgments from the difficulty or inability to retrieve requested examples (e.g., Schwarz et al., 1991), it would be better to find a way to exclude less participants. For example, three of the requested examples in Study 2 did receive a high response rate of over 90% (promotion: “Describe what the company does to provide employees with opportunities for growth”; prevention: “Describe what the company does to monitor fulfillment of the duties and responsibilities among the employees”; control: “Describe the last staff-meeting subject and its course”). Future studies need to identify more examples like this that would increase the likelihood that participants could retrieve all of the requested examples.

Next, Study 3 used a cross-sectional study design to test mediation hypothesis. Hence, causal inferences, although derived from theory and consistent with prior findings in the literature, must be made with caution. An alternative explanation would argue for a reverse relationship; i.e., individuals high in burnout may be more prone to perceiving their environment as incongruent with their needs and preferences. Future studies could address this issue by using a longitudinal design, wherein burnout would be examined as a process that develops gradually as a result of accumulated misfit strain. Hence, one could hypothesize that longer employment period only amplifies the obtained pattern of results. This research model is in line with a newly-formulated proposal to study a more dynamic model of job burnout that exposes how burnout progresses over time (Bakker and Costa, 2014).

Regulatory fit theory argues that, in addition to the *feeling right* or *feeling wrong* mechanisms, fit strengthens engagement and gives individuals a sense of control over the situation. Multiple studies to date have linked regulatory fit with task (e.g., Förster et al., 2001) or message (e.g., Pierro et al., 2013) engagement. Similarly, one could expect regulatory fit at person-organization level to have consequences for work engagement: the intensity of vigor, dedication, and absorption (Schaufeli and Bakker, 2003) that employees experience in their workplace. These consequences predicted by regulatory fit theory need to be examined in the future.

It should also be noted that regulatory fit can affect individuals differently, depending on the importance that they assign to their work. Namely, in a series of studies Avnet et al. (2013) showed that under low involvement, fit increases the positivity of feelings from a direct transfer of feeling right, making positive evaluations more positive and negative evaluations less negative. Under high involvement, on the other hand, it strengthens the original evaluations, thereby intensifying positive evaluations and also intensifying negative evaluations. For organizational studies the involvement can result from the way employees treat their work: as a calling, as a career, or as just a way to make money (Wrzesniewski et al., 1997). In that sense, it is possible that when employees perceive their job as a calling (high involvement condition), misfit would make them feel wrong about whatever else about their job was still positive to them, thereby deintensifying the positive aspects of their job. And for those low involvement employees who consider their job as just a way to make money, *feeling wrong* from misfit would directly transfer negativity to their job. These possibilities should be tested in future research.

It should also be noted that the employees are not just passive recipients of the organizational reality, but can *design* their jobs when they experience a mismatch between their current situation and their needs (Wrzesniewski and Dutton, 2001; Tims and Bakker, 2010). *Job crafting* is an initiative of employees, which is aimed at changing the job to better match their preferences (Tims and Bakker, 2010). These changes may relate to distinct aspects of the work: professional tasks, relationships in the workplace, and cognitions about the job (Wrzesniewski and Dutton, 2001). Consequently, one may argue that regulatory non-fit should lead to more intentions to craft one's job, which would predict lower misfit in weeks following actual job crafting behaviors. Moreover, Brenninkmeijer and Hekkert-Koning (2015) showed that promotion focus was positively associated with job crafting in the form of seeking resources and challenging demands (approaching gains), while prevention focus was associated with reducing hindering demands (avoiding losses). Building upon this we propose that regulatory misfit mobilizes individuals toward crafting contingent upon their preferences. This proposition should also be tested in future research.

## Concluding comment

In sum, regulatory focus and regulatory fit theories offer new insights on person-organization fit. In this research we propose a new level of fit, concerning the relation between employees' personal self-regulatory focus and the regulatory focus of their organizational climate. The experience of regulatory fit provides a mechanism concerning how individuals can transfer positive feelings from fit and negative feelings from non-fit to judgments about fairness in organizational conduct. Importantly, the negative consequences of the lack of fit relate not only to procedural justice perceptions, but also to burnout symptoms, demonstrating the significance of regulatory fit to individuals.

## Ethics statement

This study was carried out in accordance with the recommendations of Departmental Ethics Committee, SWPS University of Social Sciences and Humanities, Faculty in Sopot, with written informed consent from all subjects. All subjects gave written informed consent in accordance with the Declaration of Helsinki. The protocol was approved by the Departmental Ethics Committee, SWPS University of Social Sciences and Humanities, Faculty in Sopot.

## Author contributions

MR, SR, and ETH: Designed research; Performed research; Analyzed data and Wrote the paper.

### Conflict of interest statement

The authors declare that the research was conducted in the absence of any commercial or financial relationships that could be construed as a potential conflict of interest.

## Footnotes

^1^For instance, in task “Recall and describe an event when your organization promoted creativity among its employees” an example of a deleted response was “Things like that do not happen in my workplace.”

^2^All *p*s < 0.001 (Least Significant Difference test).

^3^Lower Level Confidence Intervals for α = 0.05.

^4^Upper Level Confidence Intervals for α = 0.05.

^5^Marginally, *p* = 0.06.

^6^Marginally, *p* = 0.07.

^7^*p* = 0.054.

## Appendix A

### Organizational regulatory focus scale (orfs; Roczniewska et al., 2016) validation process

First, we developed 10 items describing organizational climate from the perspective of a promotion orientation (focus on maximizing and seeking opportunities; striving for ideals and aspirations; growth and development as values; promoting challenges and creativity among employees) and a prevention orientation (focus on monitoring and avoiding losses; fulfillment of duties and responsibilities; safety and security as values; following procedures and codes of conduct). The content validity was confirmed by means of competent judges' ratings.

An Exploratory Factory Analysis (*N* = 655) demonstrated a two-factors solution with all factor loadings between 0.42 and 0.88. The solution was vindicated in a Confirmatory Factor Analysis using Mplus software on another sample (*N* = 691) with RMSEA = 0.078 (90% C.I.: 0.067–0.090), TLI = 0.915, CFI = 0.938, demonstrating a considerably better fit than a 1-factor solution. The Cronbach's Alpha coefficient for both scales is acceptable (Nunnally and Bernstein, 1994): α = 0.90 for promotion and α = 0.80 for prevention.

In validation studies using RFQ-proverb items (van Stekelenburg, 2006) as companies' mottos, we demonstrated that promotion-related slogans correlate significantly stronger with promotion than prevention climate, whereas the reverse was true for prevention-related slogans. Next, promotion climate, but not prevention climate, was significantly positively correlated with organization's innovation; and prevention climate, but not promotion climate, was related to bureaucracy level, both tested with Zeitz's scales (Zeitz, 1984). In another validation study, the level of organizational promotion was significantly higher in an innovative co-working organization than the level of prevention, whereas the opposite was true for a local government institution (city hall). In a criterion validity study, we also demonstrated that the scale significantly predicts company's decision-making strategies in a sunk-cost dilemma; specifically, a promotion climate is related to eagerness whereas a prevention climate is related to vigilance.

## Appendix B

**Table B1:** Means (*M*), standard deviations (*SD*), and zero-order correlations between variables in Study 1.

	**Descriptives**		**Correlations**			
	***M***	***SD***	**1**	**2**	**3**	**4**
1. Employee promotion	3.88	0.63	–			
2. Employee prevention	4.40	0.53	0.47^***^	–		
3. Organizational promotion	3.42	0.98	0.31^***^	0.19^**^	–	
4. Organizational prevention	3.87	0.71	0.25^***^	0.37^***^	0.54^***^	–
5. Procedural justice	3.32	0.77	0.31^***^	0.08	0.63^***^	0.43^***^

*N = 294*.

*** *p < 0.001*;

** *p < 0.01*.

## Appendix C

**Table C1:** Means (*M*), standard deviations (*SD*), and zero-order correlations between variables in Study 3.

	**Descriptives**		**Correlations**					
	***M***	***SD***	**1**	**2**	**3**	**4**	**5**	**6**
1. Employee promotion	3.69	0.66	–					
2. Employee prevention	4.14	0.61	0.36^***^	–				
3. Organizational promotion	3.38	1.00	0.20^***^	0.03	–			
4. Organizational prevention	3.48	0.81	0.12^*^	0.34^***^	0.43^***^	–		
5. Procedural justice	3.15	0.93	0.11^*^	−0.04	0.71^***^	0.39^***^	–	
6. Exhaustion	2.29	0.57	−0.16^**^	0.04	−0.44^***^	−0.18^***^	−0.44^***^	–
7. Disengagement	2.25	0.58	−0.22^***^	−0.08	−0.56^***^	−0.31^***^	−0.49^***^	0.68^***^

*N = 374*.

*** *p < 0.001*;

** *p < 0.01*;

* *p < 0.05*.
